# Mid-Air Tactile Sensations Evoked by Laser-Induced Plasma: A Neurophysiological Study

**DOI:** 10.3389/fnins.2021.733423

**Published:** 2021-10-01

**Authors:** Hyung-Sik Kim, Kyu Beom Kim, Je-Hyeop Lee, Jin-Ju Jung, Ye-Jin Kim, Sung-Phil Kim, Mi-Hyun Choi, Jeong-Han Yi, Soon-Cheol Chung

**Affiliations:** ^1^Department of Biomedical Engineering, BK21 Plus Research Institute of Biomedical Engineering, School of ICT Convergence Engineering, College of Science and Technology, Konkuk University, Chungju-si, South Korea; ^2^Department of Biomedical Engineering, Ulsan National Institute of Science and Technology, Ulsan, South Korea

**Keywords:** pulsed laser, plasma, tactile stimulation, mid-air, EEG

## Abstract

This study demonstrates the feasibility of a mid-air means of haptic stimulation at a long distance using the plasma effect induced by laser. We hypothesize that the stress wave generated by laser-induced plasma in the air can propagate through the air to reach the nearby human skin and evoke tactile sensation. To validate this hypothesis, we investigated somatosensory responses in the human brain to laser plasma stimuli by analyzing electroencephalography (EEG) in 14 participants. Three types of stimuli were provided to the index finger: a plasma stimulus induced from the laser, a mechanical stimulus transferred through Styrofoam stick, and a sham stimulus providing only the sound of the plasma and mechanical stimuli at the same time. The event-related desynchronization/synchronization (ERD/S) of sensorimotor rhythms (SMRs) in EEG was analyzed. Every participant verbally reported that they could feel a soft tap on the finger in response to the laser stimulus, but not to the sham stimulus. The spectrogram of EEG evoked by laser stimulation was similar to that evoked by mechanical stimulation; alpha ERD and beta ERS were present over the sensorimotor area in response to laser as well as mechanical stimuli. A decoding analysis revealed that classification error increased when discriminating ERD/S patterns between laser and mechanical stimuli, compared to the case of discriminating between laser and sham, or mechanical and sham stimuli. Our neurophysiological results confirm that tactile sensation can be evoked by the plasma effect induced by laser in the air, which may provide a mid-air haptic stimulation method.

## Introduction

Most haptic interfaces require physical contact to the body to evoke tactile sensations in users. Actuators in haptic interfaces produce physical energy that is mediated by solid substances to the skin, stimulating relevant mechanoreceptors to evoke tactile sensation. Yet, energy transmission through solid media often limits application of haptic devices to use in virtual environments. To address this issue, a number of studies have developed non-contact means of delivering stimulus energy through the air with no explicit solid media. One of the mid-air tactile stimulation method is to evoke tactile sensation *via* virtual lumps modulated by a distant air-jet display (Gwilliam et al., 2013; Sodhi et al., 2013). Other types of mid-air haptic interfaces include tactile display based on focused ultrasound (Jun et al., 2015; Yang et al., 2021). A sufficiently strong acoustic force induced by the focused ultrasound can evoke tactile sensation on the skin. The mid-air ultrasound tactile display has been continuously improved to now provide three-dimensional (3D) virtual haptic feedback (Kim et al., 2015).

Current mid-air tactile displays, however, suffer from a major drawback such that they are only working within a limited space due to significant energy attenuation over a good distance. Alternatively, laser can overcome such shortcomings with a relatively long energy travel distance if it is possible to evoke tactile sensation using laser. Recent work by our research group has proven that a thermoelastic effect induced by direct laser pulse irradiation to the skin indeed evokes tactile sensation in humans (Lee et al., 2015, 2016). In this work, a physical experiment followed by perceptual and neurophysiological experiments to evaluate human perceptual and neural responses to laser-induced tactile stimulation revealed that innocuous tactile sensation was reportedly perceived along with event-related (de)synchronization [ER(D)S] of sensorimotor rhythms (SMRs) of electroencephalography (EEG) that typically reflects tactile sensory information processing (Long et al., 2014; Sho et al., 2019). A putative explanation here is that a transient thermoelastic wave induced by laser with a certain pulse width might generate a sudden mechanical blast in the skin to activate mechanical receptors.

Although direct laser pulse irradiation to the skin could evoke tactile sensations, tactile percepts from it were only limited to pricking, touching, stinging, and warmness. Also, the perceived senses of direct laser stimulation significantly varied across individuals. Consequently, using an intermediate elastic material between laser irradiation and the skin was proposed as an alternative haptic interface (Singh et al., 2014). This indirect tactile stimulation used the same thermoelastic effect by laser but irradiated laser to an elastic medium attached to the skin. Then, the thermoelastic effect occurred inside the medium, subsequently yielding physical changes in the medium that in turn mechanically stimulated the skin. A simulation study suggested that instantaneous absorption of a laser pulse generates transient bending stress, which in turn causes deformation and vibration of the elastic medium. The study demonstrated that humans could perceive a tactile sensation similar to a short tap on the finger in response to the indirect laser irradiation (Lee et al., 2015, 2016).

However, this mid-air stimulation technique suffers from an inevitable disadvantage that one should always wear an elastic medium on the skin to generate tactile stimulation. To overcome this limitation, the present study proposes a novel way to provide tactile sensation to the skin using laser without elastic media. Our approach is based on laser plasma shock wave generated in the air by laser irradiation (Yu-Zhu et al., 2007). When an ultrashort and high intense pulsed laser is focused in the air, the laser-induced plasma occurs (Bundschuh et al., 2005; Schwarz et al., 2010). This is one of the optical breakdown phenomena where optical energy is absorbed in the air, and mechanical energy is generated by local thermal expansion. Even in the nanosecond and femtosecond time of pulsed laser, a shock wave accompanied by a burst noise is generated for hundreds of microseconds (Conesa et al., 2004; Schwarz et al., 2010). Thus, we hypothesize that mechanical pressure from the shock wave can be delivered to the skin through air to evoke tactile sensation.

To verify that laser plasma tactile stimulation is perceived as a tactile stimulation, we examine the neurophysiological responses to this stimulation in comparison with those to a simple mechanical stimulus. In particular, we analyze ERD and ERS of SMRs of EEG evoked by laser plasma stimulation over the sensorimotor area of the human brain. SMRs consisting of mu and beta rhythms decrease in magnitude (ERD) after perceiving tactile stimuli, followed by increases in magnitude (ERS) of beta rhythms (Long et al., 2014; Sho et al., 2019). We assume that ERD/S of SMRs would be manifested in response not only to a mechanical stimulus but also to the laser plasma stimulus if the laser plasma stimulation can evoke tactile sensation.

## Materials and Methods

### Laser Plasma Phenomena

Ultrashort and high intense pulsed laser is instantaneously focused in the air, and the energy of the small region becomes over high energy (>10 MW); the laser-induced plasma is generated (Conesa et al., 2004; Qin and Attenborough, 2004; Bundschuh et al., 2005; Schwarz et al., 2010). The plasma expanded because of the collapse of the medium in the air after follows the vapor occurs. These vapors are ionized and are present in quantum, neutron, and electron states. These materials collide with each other and instantly become a local high temperature state. Through this process, a high temperature is reached, and plasma is formed by the laser beam. The lifetime of the plasma through the collapse and ionization of the atoms is considerably longer than the irradiation time of the pulsed laser. In addition, shock waves accompanied by flash and a single burst noise are generated simultaneously. Scintillation is determined by the type of medium in which the laser is focused, and the magnitude of shock wave is proportional to the energy of the laser (Qin and Attenborough, 2004; Hosoya et al., 2013). As most of the energy is consumed as heat at the initial location where the plasma is generated and converted into shock waves and acoustic sound, heat is not transferred through the space to the ambient environment (Conesa et al., 2004; Qin and Attenborough, 2004; Bundschuh et al., 2005; Schwarz et al., 2010).

### Participants

Fourteen adult volunteers participated (six females, Koreans, 23.15 ± 1.79 years old; and eight males, Koreans, 24.54 ± 1.27 years old), and all participants agreed to the content of the experiments. The experiment was approved by the Ulsan National Institute of Science and Technology institutional review board (UNISTIRB-15-16-A).

### Laser Stimulation Setup

**FIGURE 1:**
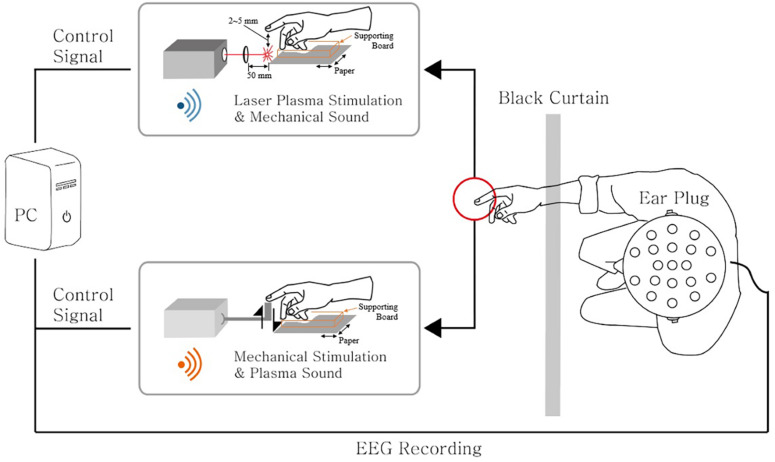
An illustration of the experimental setup. Laser plasma stimulation along with the auditory mechanical sound was provided to the subject’s right index finger. Also, mechanical stimulation along with the auditory plasma sound was provided to the same finger location. The 21-channel electroencephalography (EEG) signals were recorded simultaneously. The subject’s view was blocked by the black curtain to inhibit the visual effects of the stimuli. An ear plug was used to reduce the sound effects induced by stimulations. The laser device and the mechanical actuator were triggered by the control signal generated from the PC.

Figure 1 represents the schematic diagram of the experimental environment. M-NANO 40–100 mJ Nd:YAG laser is used to provide laser plasma tactile stimulation at the index finger. PCX lens of focal length 50.0 mm and diameter 25.0 mm was used to make plasma of the laser under the subject’s index finger. When a trigger is given to the device, flashlight and high tone sound occur. Black curtain is used to prevent the flashlight. Subject’s wrist, palm, and index finger are supported using Styrofoam board. The distance between the end point of the finger and the position that laser plasma happens was controlled with papers under the supporting board to provide distinct tactile sensation, ensuring the subject does not take laser stimulus directly at the index finger. The average distance where all subjects clearly felt the tactile sensation was 2–5 mm, and there was no temperature change or heat sensation on the fingers.

### Task Procedure

**FIGURE 2:**
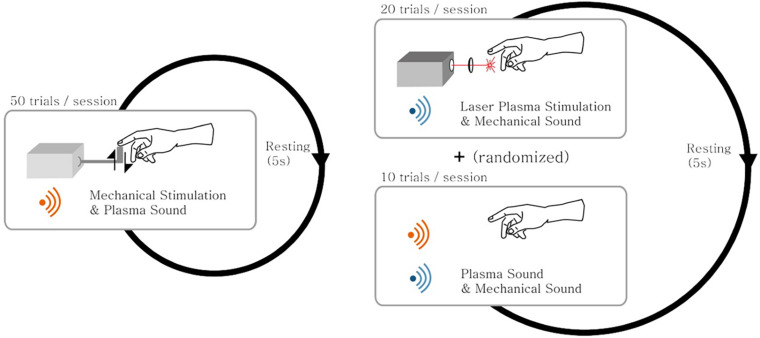
A schematic diagram of the experimental design. The experiment consisted of both the mechanical stimulation session and the laser plasma stimulation session mixed with sham stimulation. (Left) In the mechanical stimulation session, a total of 50 trials of mechanical stimulation along with the auditory laser plasma sound were given to the subjects. (Right) In the laser plasma stimulation session, a total of 20 trials of laser plasma stimulation along with the mechanical stimulation sound were given to the subjects. In addition, a total of 10 trials of sham stimulation with a mixed sound of both laser plasma and mechanical sounds interspersed with the laser stimulation trials in a random fashion. The subjects rested for 5 s between successive trials of stimulation.

Figure 2 presents the experimental design. It consists of the first two sessions of mechanical stimulation where each session had 50 trials, and the next five sessions consisted of sham and laser plasma tactile stimulations where each session had 10 sham stimulation trials and 20 laser plasma tactile stimulation trials were randomly given. Thus, subjects had 100, 100, and 50 times of mechanical stimulation, laser plasma tactile stimulation, and sham stimulation, respectively.

Mechanical stimulation and sham stimulation are given to compare the brain response with that of laser stimulation. Mechanical stimulus was given with Dynamixel RX-64 robot actuator. It rotates the wheel attached next to the body of the actuator, and energy is transferred to a Styrofoam board connected to the wheel. The board touches the end of the index finger and returns to the original position with mechanical sound. Revolution per minute of the actuator was controlled. Sham stimulation was given as sounds of laser plasma and actuator at the same time (Figure 1).

Through the pilot experiment, the output energy of the laser was set at approximately 70 mJ and the rotation speed of the wheel at approximately 450 revolution/min so that all subjects could clearly and perceptually detect both laser stimulation and mechanical stimulation without pain. To minimize the effect of sound resulting from the actuator and laser plasma to neural response, tactile stimulation and counterpart sound are presented simultaneously. For example, when the laser stimulation is presented, the recorded sound of the actuator is played at the same time. Also, all the subjects were required to wear earplugs to prevent the sounds distracting the subject’s attraction during the experiments. M-NANO device is controlled with C# program, and the Dynamixel actuator is controlled with MATLAB.

### Electroencephalography Recordings

Scalp EEG was recorded using an EEG headset with 21 dry electrodes (DSI-24, Wearable Sensing, Inc., United States). Electrodes were positioned over the head following the International 10–20 System. EEG signal was sampled at 300 Hz and initially referenced to the electrode at Pz. After recordings, each EEG signal was re-referenced to the right and left earlobes. EEG signals recorded from three channels of C3, Cz, and C4 placed over the sensorimotor area were analyzed in this study. These EEG signals were epoched from −3 to 3 s after stimulation onset and bandpass filtered from 2 to 40 Hz. Then, the subset of this epoched and filtered signal segment, from 0.5 to 2 s around the stimulation onset, was included in the data analysis. The trials including EEG amplitudes larger than 4.5 times of the standard deviation of amplitude over all the epochs were excluded. In doing so, 89.7 ± 4.12, 91.1 ± 4.63, and 44.3 ± 2.98 trials for laser, mechanical, and sham stimulation, respectively, were passed to the subsequent analysis procedure below. Because of the unexpected failure of EEG recordings during the experiment, the data of one subject were discarded, leaving the data sets of 13 subjects for subsequent analyses.

### Data Analysis

The overall data analysis procedure in this study was as follows. First, we applied short-time Fourier transform (STFT) and statistical analysis methods to epoched EEG data to identify ERD/S induced by each stimulus. The estimated power spectral density (PSD) of EEG responses was compared pairwisely between the stimuli using repeated-measures ANOVA (rmANOVA) and *post hoc* tests. Second, to assess the similarity of EEG responses between the stimuli, both a decoding analysis and a statistical distance analysis were conducted.

#### Spectral Analysis

**FIGURE 3:**
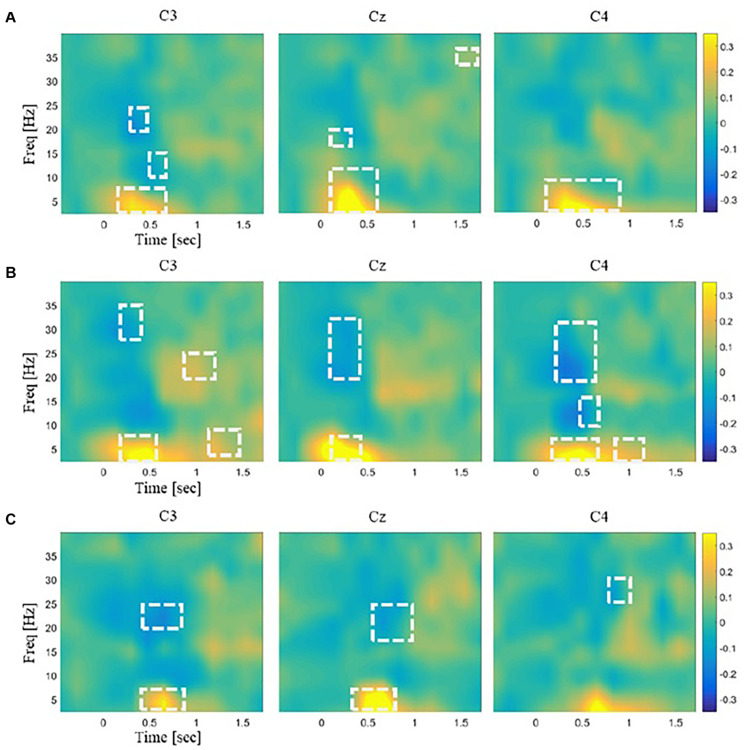
Illustrations of the time–frequency EEG responses to stimulations. The spectral power for each frequency band was normalized to the mean of the baseline period (–2,000 ms). The normalized power data were averaged across the trials for each channel and each stimulus. Time–frequency regions showing significant changes in power from baseline were marked by white dashed lines (*p* < 0.01). The color bar unit indicates the power ratio. The time–frequency EEG patterns for three somatosensory channels C3, Cz, and C4 are shown in response to panel **(A)** laser plasma stimulation, **(B)** mechanical stimulation, and **(C)** sham stimulation.

**FIGURE 4:**
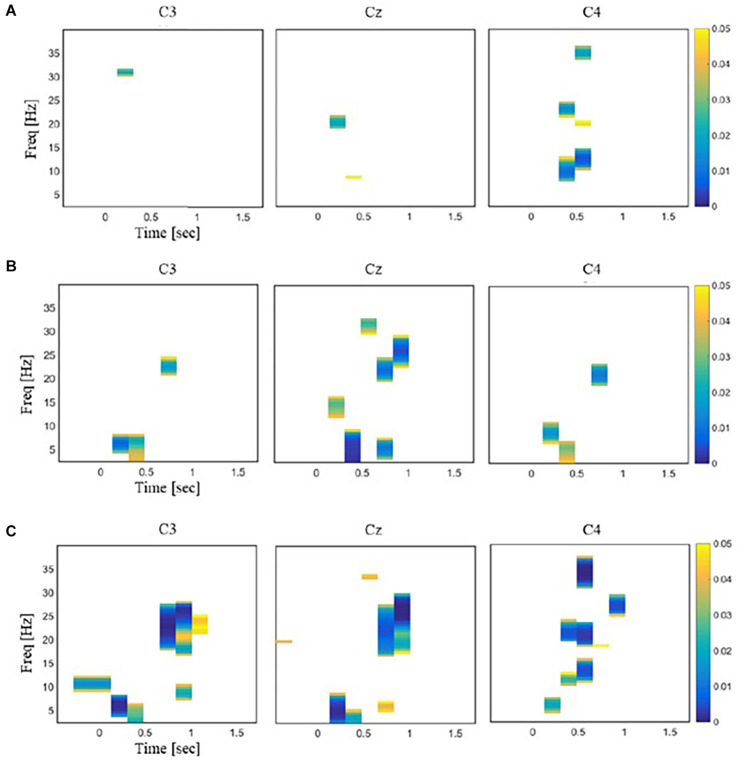
Illustrations of the time–frequency regions in EEG responses that exhibited significant differences between a pair of stimuli [repeated-measures ANOVA (rmANOVA), *p* < 0.05]. The color bar represents the *p* values with Bonferroni correction. **(A)** Comparison between laser and mechanical stimulation. **(B)** Comparison between laser and sham stimulation. **(C)** Comparison between mechanical and sham stimulation.

An EEG response to each stimulus at each channel was obtained as follows. Time-varying PSD over a single epoch was estimated using STFT with the Hanning window of 0.25 s and an overlap of 0.075 s. It generated an *N* × *M* matrix of PSD data where *N* was the number of frequency bands, and *M* was the number of windows (*N* = 65 and *M* = 12 in our analysis). This time–frequency power matrix was repeatedly constructed from all the trials and normalized by subtracting the mean of baseline values and dividing by the baseline standard deviation in each subject. The normalized power value was averaged across trials for each channel and each stimulus for every subject. If the normalized power value was greater than zero, it was marked as ERS, and if less than zero, it was marked as ERD. The statistical significance of ERD/S for each time–frequency bin was assessed by a two-tailed *t* test with a null hypothesis that the mean of normalized power values across participants was zero (*p* < 0.01; Figure 3). Statistical differences of ERD/S between the three stimuli were assessed for each channel using rmANOVA and *post hoc t* tests with Bonferroni correction (*p* < 0.05; Figure 4). All the time–frequency bins from a given channel in the range of 2–28 Hz and 0–1 s after stimulation onset were collected into an EEG feature vector for the subsequent decoding analysis. This range was chosen because the time–frequency bins showing significant differences between the stimuli were mostly observed in this range (Figure 4).

Moreover, as the length of these feature vectors was greater than the number of samples available to build decoders, a dimensionality reduction method was used to project the original feature vectors onto a new low-dimensional space. Such dimensionality reduction could also help us visualize EEG features and compare between the stimuli. We reduced dimensionality of each stimulus’ and channel’s feature vector using non-metric multidimensional scaling (NMDS) that can preserve the distance between observations in an original feature space (Michael et al., 2016). The collected feature vectors were reduced to a low dimensionality by the NMDS for decoding and statistical distance analysis. To determine this low dimensionality, we continuously reduced the dimensionality of the original feature vector using the NMDS until the stress value became less than 0.1, which was generally regarded as a good fit (Hair et al., 2005). In our data, the stress value became less than 0.1 when we reduced the dimensionality to 4 or less. Hence, we chose the dimensionality equal to 4. For the visualization, the collected feature vectors were reduced to 2 dimensionality by the NMDS, which had a stress value lower than 0.2.

#### Decoding Analysis

Quadratic discriminant analysis (QDA) was used for decoding as a classifier that classified a feature vector into one of the stimulus classes (Zhang, 1997). QDA was independently built three times to classify the low-dimensional vectors projected by NMDS into one of the two classes according to each pair of the stimuli: laser versus mechanical, laser versus sham, and mechanical versus sham. For instance, if the decoding analysis compared EEG responses between the laser and sham stimuli, and NMDS projected the EEG feature vectors of every participant onto a 4D space, the input data to QDA were a set of the 4D samples where each sample corresponded to an EEG response of one of the participants to either the laser or sham stimulus. The performance of QDA was cross-validated *via* the leave-one-out method. We considered that low decoding accuracy indicated similar EEG response patterns between the compared stimuli, and high accuracy indicated the opposite case.

#### Statistical Distance Analysis

Statistical distances between the distributions of the low-dimensional EEG features, which had the same dimensionality with that used for the decoding analysis, for each pair of the stimuli at each electrode were estimated. Bhattacharyya distance measure assuming Gaussian distribution was used to quantify the similarity of probability distributions.

## Results

### Electroencephalography Responses

Spectral analysis showed significant ERD/S in the sensorimotor area (Figure 3). The mechanical and laser stimulation induced beta (20–30 Hz) ERD within 0.5 s after stimulus onset. The sham stimulation induced only beta ERD that occurred 0.5 s after onset. The channels showing significant beta ERD were C3 for the laser stimulation; C3, Cz, and C4 for the mechanical stimulation; and C3 and Cz for the sham, respectively. In addition, the mechanical stimulation induced alpha ERD (8–13 Hz) at C3. Theta (4–8 Hz) ERS was observed within 0.5 s after stimulus onset in response to the laser and mechanical stimulation and 0.5–0.8 s after onset in response to the sham stimulation at all the channels.

The numbers of time–frequency bins showing significant ERD/S were 202, 96, and 64 for the mechanical, laser, and sham stimulation, respectively. The mean sizes of the clusters, defined as the set of significant time–frequency bins adjacent to each other, were 20.9, 11.9, and 8 bins for the mechanical, laser, and sham stimulation, respectively.

**FIGURE 5:**
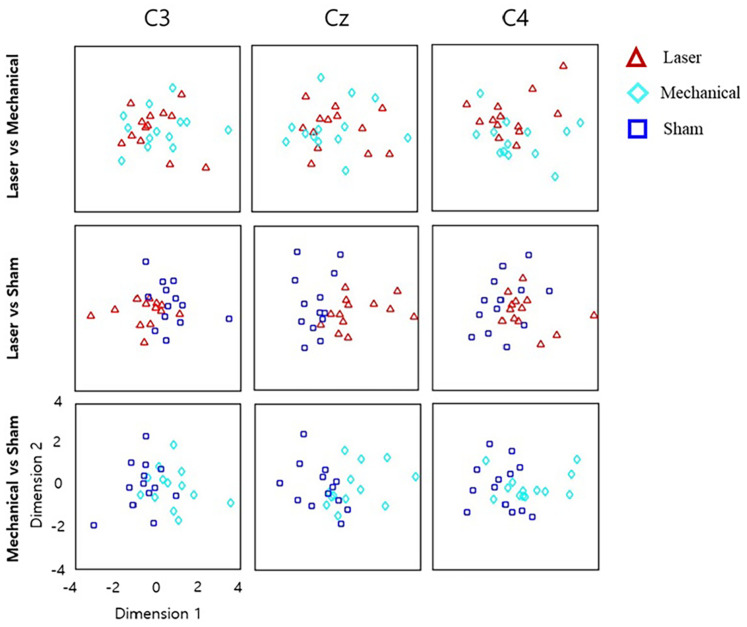
Low-dimensional representations of EEG responses to different stimuli, reduced by non-metric multidimensional scaling (NMDS). The original, high-dimensional EEG features at each channel were reduced to the two dimensional space. Each point corresponds to the 2D representation of the significant time–frequency EEG responses averaged over the trials in response to a given stimulus in each subject. Note that in the same channel, the 2D representations of EEG responses to a stimulus (e.g., laser) differed for different pairs (e.g., laser-mechanical and laser-sham) because the original time–frequency EEG responses selected for each stimulus pair varied by different statistical results (see the text). The horizontal axis denotes the first dimension reduced by NMDS, and the vertical axis denotes the second dimension.

Repeated-measures ANOVA revealed time–frequency bins at beta and theta bands that were significantly different across the three stimuli. For these bins, *post hoc t* tests showed little difference in the power values between the laser and mechanical stimulations. In contrast, *post hoc t* tests revealed large differences of the power values for the sham from those for the mechanical and laser stimulation (Figure 4). Specially, the number of time–frequency bins showing significant difference between a pair of the stimuli was 45 for mechanical versus laser, 199 for mechanical versus sham, and 103 for laser versus sham, respectively. Figure 5 illustrates an example of the 2D representations of EEG responses to each stimulus, reduced by NMDS. It was apparent from this illustration that the distributions of the representations of EEG responses to the mechanical and laser stimuli were closer to each other than those to the sham stimulus. For the following decoding and distance analyses, dimensionality of the low-dimensional space was determined to be 4.

### Decoding Outcomes and Bhattacharyya Distances

**FIGURE 6:**
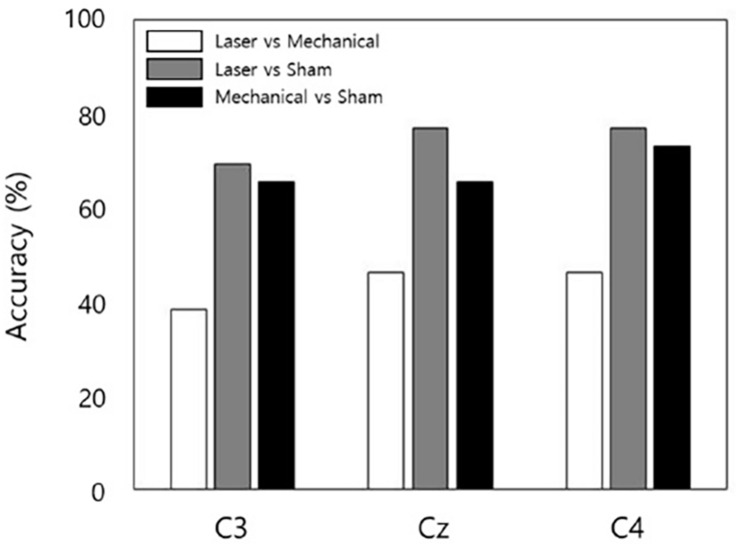
The accuracy of decoding stimulation information from EEG data for each stimulus pair and each channel. The decoding model performed binary classification by classifying EEG data into one of the two stimuli in a pair.

For channel C3, decoding accuracy of QDA was 38% between the laser and mechanical stimulation, 69% between the laser and sham, and 65% between the mechanical and sham. For channel Cz, it was 46, 77, and 65% for laser-mechanical, laser-sham, and mechanical-sham classification, respectively. For channel C4, it was 46, 77, and 73% for laser-mechanical, laser-sham, and mechanical-sham classification, respectively. In summary, the lowest classification accuracy was obtained between the mechanical and laser stimulation (Figure 6).

**FIGURE 7:**
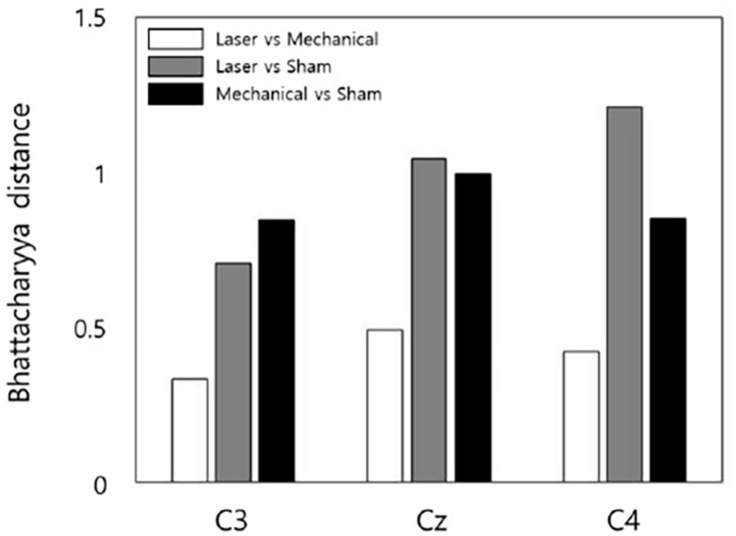
The statistical Bhattacharyya distances of EEG responses between a pair of stimuli for each channel. Shorter distances indicate more similar EEG responses to a pair of stimuli. Note that no error bar showing the variability across subjects is included here since the Bhattacharyya distance was measured using the EEG data of all subjects.

For channel C3, the shortest Bhattacharyya distance was found between the distribution of laser and mechanical stimuli, and the longest Bhattacharyya distance was found between the distributions of the mechanical and sham stimuli. For channel Cz, the shortest Bhattacharyya distance was found between the distribution of laser and mechanical stimuli, and the longest Bhattacharyya distance was found between the distributions of the laser and sham stimuli. For channel C4, the shortest Bhattacharyya distance was found between the distribution of laser and mechanical stimuli, and the longest Bhattacharyya distance was found between the distributions of the laser and sham stimuli (Figure 7).

## Discussion

The present study investigated whether the shock wave generated by laser-induced plasma could stimulate the skin to evoke tactile sensation. We demonstrated that humans could perceive a tactile sense from laser-induced plasma by measuring EEG responses to stimulation. Specifically, the power changes (ERD/S) of SMRs over sensorimotor cortical areas, which indicated the modulation of SMRs by tactile perception, were observed in response to the stimulation of laser-induced plasma. The time–frequency pattern of EEG responses to the laser stimulus was similar to that to the mechanical stimulus, but dissimilar to the sham stimulus. Moreover, two statistical analyses were conducted to quantitatively examine the time–frequency patterns of EEG. A statistical distance analysis based on the Bhattacharyya distance metric revealed that the EEG responses to the laser stimulus were closer to those to the mechanical stimulus than those to the sham stimulus. A decoding analysis using QDA revealed that it was more difficult to discriminate the EEG response patterns between the laser and mechanical stimuli than between the laser and sham stimuli. Our results thus suggest that laser-induced plasma could induce neural responses mediating tactile perception. The demonstration of mid-air tactile sensation evoked by laser-induced plasma may offer a new opportunity to develop non-contact haptic interfaces.

Laser can be focused on a distant location with high precision from a remote source as long as ∼10 m. Such long-distance stimulation can overcome limitations of current ultrasonic or air jet mid-air stimulation technologies (Gwilliam et al., 2013; Jun et al., 2015; Kim et al., 2015; Ismo et al., 2020; Yang et al., 2021). Also, tactile stimulation using laser-induced plasma does not require an explicit elastic medium attached to the skin as in the previous study (Singh et al., 2014; Lee et al., 2016), making itself available to more general applications. This mid-air tactile sensation using the laser-plasma phenomenon has to consider two distances. The first is the distance between the laser system and the plasma. Theoretically, the laser irradiation distance is infinite. However, as the laser has to be focused in mid-air using a lens to generate plasma, the distance is shortened to the level of several tens of centimeters or meters. However, it is a longer distance than the existing focused ultrasound or air jet methods. Distance control can simply increase or decrease by adjusting the focal length of the lens. However, if a long focal length of several tens of meters or more is used, plasma may not be generated. This is because the energy per unit area becomes small at the focusing location, so the plasma generation condition in mid-air cannot be sufficiently exceeded (Bundschuh et al., 2005; Schwarz et al., 2010). In this case, it can be generated by increasing the output energy of the laser system or using a pulse laser with a shorter pulse width (Conesa et al., 2004). The second is the distance between the plasma and the finger, which is the distance through which the shock wave is transmitted. In this study, the distance at which all subjects clearly perceived tactile sensation was 2–5 mm. To increase this distance, it is possible to increase the output energy of the laser system or use a laser with a pulse shorter than the pulse width of the currently used laser system (about 10 ns).

The output energy of the laser and the size of the generated shock wave increase proportionally and become saturated. In addition, the magnitude of shock waves generated by laser-induced plasma exponentially decreased when they traveled away from an initial location of plasma (Qin and Attenborough, 2004). This is due to the density of ions in space and the limited lifetime of the shock wave (Conesa et al., 2004; Schwarz et al., 2010). It implies that the maintenance of distance from the plasma location to the skin may be crucial for reliable tactile sensation. However, shock waves from laser-induced plasma are non-directional (Hosoya et al., 2013), indicating that the direction of the skin surface relative to the plasma location would be less critical, except for the beam path. The sound effect generated by plasma is currently unavoidable. Potential applications using this haptic technique should therefore consider such auditory effects. Finally, the proposed tactile stimulation method could evoke a tactile sense consistently across different individuals, which was not the case when laser was directly irradiated to the skin (Jun et al., 2015). In addition, the distance for delivering tactile sensation is relatively long, and as it elicits a mechanical tap feeling, it can be used for applications that present tactile sensation while freely moving the hand when comprised with a finger, hand, or motion-tracking device. It will be applicable to the purpose of inducing non-contact tactile feedback when a finger touches a specific location in mid-air, and the console control field that provides tactile feedback when a button drawn in the mid-air is pressed with virtual reality technology. It will also be possible to provide multipoint tactile sensation when used with devices that reflect light such as optical mirror. The time–frequency analysis of EEG responses to the stimulus by laser-induced plasma revealed ERD of mu and beta rhythms followed by ERS of beta rhythms. These ERD-ERS patterns of SMRs have been implicated in cortical responses to tactile stimulation. Stancák (2006) showed ERD of mu and beta rhythms in response to cutaneous tactile stimulation over contralateral and ipsilateral sensorimotor areas. Other studies have reported that tactile stimulation induced initial decreases followed by rebounding increases in the power of beta rhythms (Kilavik et al., 2013; Eriko and Fuminari, 2019; Mia et al., 2021). Hence, the suppression of alpha and beta rhythms followed by the rebound of beta rhythms in response to laser-induced plasma stimulation may indicate tactile information processing of the brain.

It was shown in our results that the sham stimulus consisting of the sound of laser irradiation without actual delivery of laser pulse seemed to induce the ERD of beta rhythm. But, this ERD occurred later than those induced by mechanical and laser stimuli. It remains, however, unclear how SMR modulation occurred in response to an auditory stimulus over the sensorimotor area. One plausible explanation might be related to association between tactile sensation and the auditory stimulus of laser irradiation because every time laser pulse was irradiated in the air, the sound of plasma was inevitably delivered to participants and thus likely to be synchronized with tactile sensation. Previous studies have reported that a modality other than touch can induce tactile sensation. A number of studies have shown that only observation of touch can modulate somatosensory cortical activity (Schaefer et al., 2021a,b). Other studies have shown that auditory signals could modulate tactile perception (Bresciani et al., 2005; Md Shoaibur et al., 2020), and the tactile shape information could be formed by auditory input with training (Kim and Zatorre, 2011). Thus, it might be possible that participants could perceive a tactile sense when hearing the sound of laser irradiation only. Yet, this possibility should be further investigated with more rigorous perceptual experimental tasks.

Note that there is a sample imbalance problem between the classes as the number of trials for sham stimulation was approximately half of those of other stimulations. In the current study, we did not examine this imbalance problem as our primary goal in the decoding analysis was to demonstrate that ERD/S patterns between laser plasma and mechanical stimuli were more difficult to discriminate than those between laser plasma and sham stimuli and that classification accuracy between laser plasma and sham stimuli was similar to that between mechanical and sham stimuli, indicating that ERD/S patterns of both laser plasma and mechanical stimuli were different from those of sham stimulus to a similar degree. Although imbalanced sample sizes could affect classification results, we speculate that these main classification results would not be substantially changed by rebalancing the sample size because oversampling of the sham samples, which can be a simplest way of overcoming the current imbalance problem, may improve classification performance rather than decreasing it as the classifier will be learned with more samples. On the other hand, there is no imbalance issue between laser and mechanical stimuli so that low classification accuracy will remain unchanged (Figure 6). Therefore, possible solutions to address the imbalance problem would not change our main classification results. However, the sample imbalance is still a limitation of the present study and should be addressed properly in the future work. The current study is limited by the fact that the participants did not perform any behavioral task in the perception of tactile stimuli. Although it was confirmed during the experiment that every participant could perceptually detect a tactile stimulus when laser-induced plasma stimulation was delivered to the skin, a more systematic psychophysical experiment can shed light on detailed properties of human perceptual responses to tactile stimulation by laser-induced plasma. Hence, our follow-up study will verify psychophysical studies on the perception of laser-induced plasma tactile stimuli as well as subjective reports of more precise description of the type of tactile sensations they evoke. Additionally, we did not examine the effect of various laser parameters on neural responses and perception, such as pulse energy, repetition rate, and pulse width (Lee et al., 2016). We will investigate whether laser-induced plasma stimulation can deliver different tactile sensations through parameter adjustment.

## Data Availability Statement

The raw data supporting the conclusions of this article will be made available by the authors, without undue reservation.

## Ethics Statement

The studies involving human participants were reviewed and approved by Ulsan National Institute of Science and Technology Institutional Review Board (UNISTIRB-15-16-A). The patients/participants provided their written informed consent to participate in this study.

## Author Contributions

H-SK, S-PK, and S-CC: study conception and design and review the manuscript. KK, J-HL, J-JJ, and Y-JK: acquisition of data. H-SK, S-PK, M-HC, and J-HY: analysis and interpretation of data. H-SK and S-PK: drafting of the manuscript. All authors contributed to the article and approved the submitted version.

## Conflict of Interest

The authors declare that the research was conducted in the absence of any commercial or financial relationships that could be construed as a potential conflict of interest.

## Publisher’s Note

All claims expressed in this article are solely those of the authors and do not necessarily represent those of their affiliated organizations, or those of the publisher, the editors and the reviewers. Any product that may be evaluated in this article, or claim that may be made by its manufacturer, is not guaranteed or endorsed by the publisher.
